# Selected Essential Oils Modulate Ethidium Bromide Accumulation and Exhibit Antibacterial Activity Against Multidrug-Resistant *Escherichia coli* and *Klebsiella pneumoniae*

**DOI:** 10.3390/plants15172587

**Published:** 2026-08-25

**Authors:** Edit Ormai, Nikoletta Szemerédi, Béla Kocsis, Margita Szilágyi-Utczás, Tamás Ollmann, Gabriella Spengler, Györgyi Horváth, Viktória Lilla Balázs

**Affiliations:** 1 Department of Pharmacognosy, Faculty of Pharmacy, University of Pécs, 7624 Pécs, Hungary; ormai.edit@gytk.pte.hu (E.O.); balazs.viktoria@gytk.pte.hu (V.L.B.); 2 Department of Medical Microbiology Educational and Research Center, Albert Szent-Györgyi Medical School, University of Szeged, 6725 Szeged, Hungary; szemeredi.nikoletta@med.u-szeged.hu (N.S.); spengler.gabriella@med.u-szeged.hu (G.S.); 3 Department of Medical Microbiology and Immunology, Medical School, University of Pécs, 7624 Pécs, Hungary; kocsis.bela@pte.hu; 4 Center for Sports Nutrition Science, Hungarian University of Sports Science, 1123 Budapest, Hungary; utczas.margita@tf.hu; 5 Department of Physiology, Medical School, University of Pécs, 7622 Pécs, Hungary; tamas.ollmann@aok.pte.hu

**Keywords:** *Citrus limon* (L.) Osbeck, efflux-associated processes, *Eucalyptus radiata* Sieber ex DC., Gram-negative bacteria, healthcare-associated infection, *Melaleuca alternifolia* (Maiden & Betche) Cheel, *Pinus sylvestris* L.

## Abstract

The increasing prevalence of multidrug-resistant (MDR) Gram-negative bacteria has stimulated the search for plant-derived antimicrobials with alternative modes of action. This study investigated whether chemically distinct essential oils (EOs) differ in their antibacterial effects and their ability to modulate bacterial efflux-associated processes in clinically relevant MDR pathogens. The chemical compositions of tea tree (*Melaleuca alternifolia*), lemon (*Citrus limon*), Scots pine (*Pinus sylvestris*), and *Eucalyptus radiata* EOs, as well as their blend, were characterized by gas chromatography–mass spectrometry (GC-MS). Terpinen-4-ol, limonene, *α*-pinene, and eucalyptol were identified as major constituents. Antibacterial activity against *Escherichia coli* and *Klebsiella pneumoniae* strains with different resistance profiles was evaluated by broth microdilution, while effects on efflux-associated processes were assessed using an ethidium bromide accumulation assay. The EOs showed strain-dependent antibacterial effects, with *E. coli* being more susceptible than *K. pneumoniae*. *E. radiata* showed the lowest MIC against *E. coli* 130063 (0.312 mg/mL), whereas no EO inhibited the *K. pneumoniae* strains at concentrations below 5 mg/mL. The highest RFI values were observed for lemon oil against *K. pneumoniae* 33443 (1.41) and the EO blend against *E. coli* 128673 (1.27). These findings support further investigation of selected EOs as complementary antimicrobial agents.

## 1. Introduction

Hospital-acquired infections (HAIs), also referred to as nosocomial infections, are defined as infections developing at least 48 h after hospital admission, within three days after discharge, or within 30 days following surgery [1]. HAIs remain a major global public health challenge, contributing substantially to patient morbidity, prolonged hospitalization, increased antimicrobial consumption, and rising healthcare costs [2]. Their transmission occurs through direct contact between patients and healthcare workers, contaminated medical equipment or environmental surfaces, and, in certain cases, airborne dissemination [3]. In addition, biofilm formation on indwelling medical devices facilitates persistent infections by protecting microorganisms from both host immune responses and antimicrobial therapy [4]. Patients undergoing invasive procedures, prolonged hospitalization, or broad-spectrum antibiotic treatment are particularly susceptible to HAIs [5].

A major concern in modern healthcare is the increasing prevalence of antimicrobial-resistant (AMR) pathogens associated with HAIs [6,7]. AMR enables microorganisms to survive exposure to antimicrobial agents through genetic mutations or the acquisition of resistance determinants [8,9]. The continued spread of AMR compromises routine medical interventions, limits therapeutic options, and is considered one of the greatest threats to global health [10]. Within the spectrum of AMR, multidrug-resistant (MDR) organisms represent an especially concerning category that warrants focused discussion [11].

The present study focused exclusively on MDR Gram-negative bacteria because they include several of the World Health Organization’s highest-priority bacterial pathogens associated with antimicrobial resistance [12]. Multidrug-resistant *Escherichia coli* and *Klebsiella pneumoniae* (Enterobacterales) have become increasingly prevalent causes of healthcare-associated infections, particularly in intensive care units [13]. These pathogens are responsible for a wide range of severe infections, including urinary tract infections, bloodstream infections, pneumonia, and ventilator-associated pneumonia, particularly in immunocompromised patients [14]. In contrast, Gram-positive pathogens were not included in the present study, as the primary objective was to investigate clinically relevant Gram-negative isolates in which resistance mechanisms, especially those mediated by the AcrAB-TolC efflux system, play a major role. Furthermore, such MDR Gram-negative clinical isolates were available from our hospital setting, whereas comparable Gram-positive isolates were not collected during the study period.

One of the major contributors to multidrug resistance in Gram-negative bacteria is multidrug efflux pumps, which export structurally diverse antimicrobial compounds from bacterial cells, thereby reducing intracellular drug accumulation and treatment efficacy [15]. Among Enterobacterales, the AcrAB-TolC efflux system represents one of the best-characterized resistance mechanisms and contributes not only to antibiotic resistance but also to bacterial adaptation and persistence under environmental stress [16]. Consequently, inhibition or modulation of bacterial efflux systems has emerged as a promising complementary strategy to restore antimicrobial susceptibility and combat multidrug resistance.

Natural products have attracted considerable attention as potential sources of antimicrobial and resistance-modifying agents. Plants produce a wide variety of secondary metabolites that protect them against microbial attack and environmental stress [17,18].

Among these metabolites, essential oils (EOs) are complex mixtures of volatile compounds, primarily terpenes, terpenoids, phenylpropanoids, and other bioactive constituents extracted from different plant organs [19,20,21]. Numerous studies have demonstrated that EOs possess antibacterial activity through multiple mechanisms, including disruption of bacterial membranes, inhibition of enzyme activity, interference with quorum sensing, and prevention of biofilm formation [22]. More recently, increasing attention has been directed toward their ability to interfere with bacterial resistance-associated mechanisms, particularly efflux-mediated transport. Such multimodal mechanisms make EOs attractive candidates as complementary antimicrobial agents and potential tools for reducing the persistence and transmission of MDR pathogens in healthcare environments.

Although the antibacterial properties of numerous EOs have been extensively investigated, considerably less attention has been paid to their ability to interfere with bacterial resistance mechanisms, particularly efflux-mediated resistance in clinically relevant multidrug-resistant Gram-negative pathogens. Furthermore, comparative studies evaluating chemically distinct commercial EOs under identical experimental conditions remain scarce. We therefore hypothesized that EOs with distinct chemical compositions differ in their ability to interfere with bacterial efflux-associated processes and that such interference may contribute to their antibacterial effects against multidrug-resistant *E. coli* and *K. pneumoniae*. Accordingly, the aims of the present study were: (1) to characterize the chemical composition of selected commercially available EOs and their blend using GC-MS; (2) to evaluate their antibacterial activity against clinically relevant MDR *E. coli* and *K. pneumoniae* isolates; and (3) to investigate their ability to modulate bacterial efflux-associated processes using an EB accumulation assay. By integrating chemical characterization with antibacterial and functional analyses in well-characterized MDR clinical isolates, this study aimed to provide new insights into the relationship between EO composition and resistance-associated bacterial responses.

## 2. Results

### 2.1. Chemical Composition of EOs

The semi-quantitative volatile compositions of the EOs used in this study are presented in the Supplementary Material (Table S1). Tea tree EO contained terpinen-4-ol (38.60%) as its predominant constituent, followed by *p*-cymene (26.88%) and eucalyptol (6.01%) as minor components. In lemon oil, limonene was the dominant constituent (64.80%), followed by *β*-pinene (13.67%). Scots pine EO showed *α*-pinene (38.66%) as the predominant compound, along with *β*-pinene (15.24%), *δ*-3-carene (11.32%), and limonene (8.55%). *Eucalyptus radiata* oil was primarily characterized by eucalyptol (75.05%), with *α*-terpineol (8.09%) present as the second most abundant constituent. The EO blend showed eucalyptol as the main component (37.71%), followed by limonene (19.48%), *α*-pinene (11.68%), and *β*-pinene (5.38%). Although the chemical profile of the blend was determined analytically, the exact quantitative composition could not be reported in accordance with the agreement of the manufacturer.

### 2.2. Antimicrobial Activity (MIC)

The antimicrobial activity of the tested EOs was assessed by determining the minimum inhibitory concentrations (MICs) of all isolates. The MIC values showed substantial variation across the strains. For some EO–bacterium combinations, the MIC could not be determined within the tested concentration range, indicating that it exceeded the highest concentration tested (5.000 mg/mL). The results are shown in Table 1.

Among the tested EOs, *E. radiata* showed the lowest MIC values against the *E. coli* isolates (0.625 and 0.312 mg/mL). The EO blend showed MICs of 1.250 and 0.625 mg/mL, while tea tree EO showed an MIC of 1.250 mg/mL against both *E. coli* isolates. Scots pine showed weaker inhibition (2.500–5.000 mg/mL), and lemon EO did not inhibit either *E. coli* strain within the tested concentration range. No EO inhibited the *K. pneumoniae* isolates at concentrations below 5.000 mg/mL. These MIC results provided a basis for subsequent analysis of EO-mediated changes in EB accumulation.

### 2.3. Effects of EOs on Ethidium Bromide (EB) Accumulation

The effects of EOs on efflux-associated processes in *E. coli* and *K. pneumoniae* strains were assessed by measuring the intracellular accumulation of EB, a substrate of bacterial efflux pumps. Increased intracellular EB accumulation was considered indicative of interference with efflux-associated processes. Bacterial cultures were treated with different EO concentrations (Table 2) and compared with positive controls to determine whether the oils could enhance intracellular EB accumulation. For EO–bacterium combinations in which the MIC exceeded the highest concentration tested (>5.0 mg/mL), 2.5 mg/mL was used as an operational test concentration corresponding to half of the highest concentration examined, rather than a confirmed MIC/2. Known efflux pump inhibitors (CCCP, PMZ, and PA-βN) were included as positive controls to benchmark the EB accumulation assay [23,24,25].

**Figure 1:**
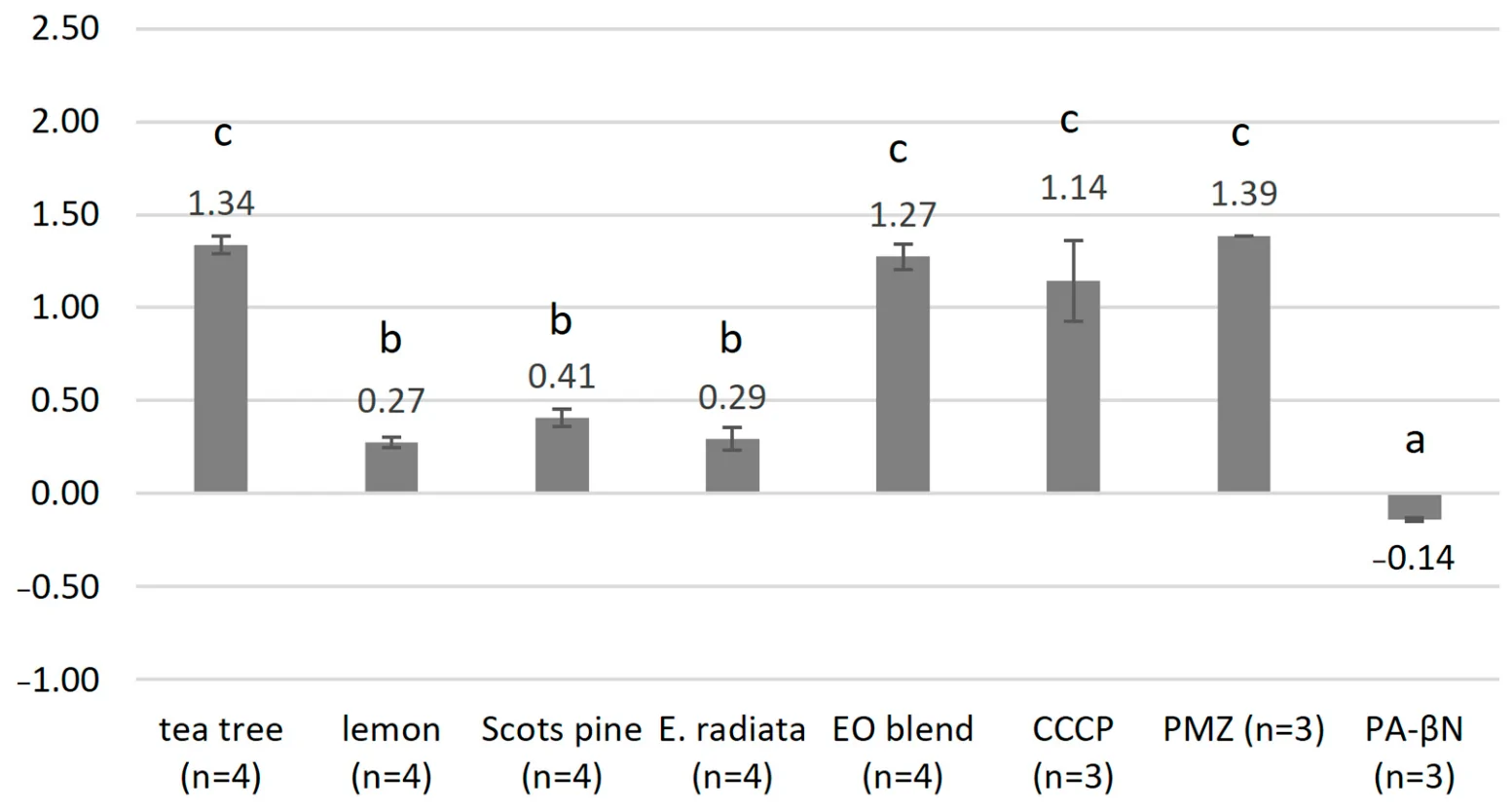
Relative fluorescence index (RFI) values of the EOs and controls in *E. coli* 128673. Bars represent mean RFI values, with error bars indicating the standard error of the mean (SEM); individual *n* values are indicated below the treatment labels. Different lowercase letters indicate statistically significant differences among treatments according to Tukey’s post hoc test (*p* < 0.05); treatments sharing at least one letter are not significantly different.

**Figure 2:**
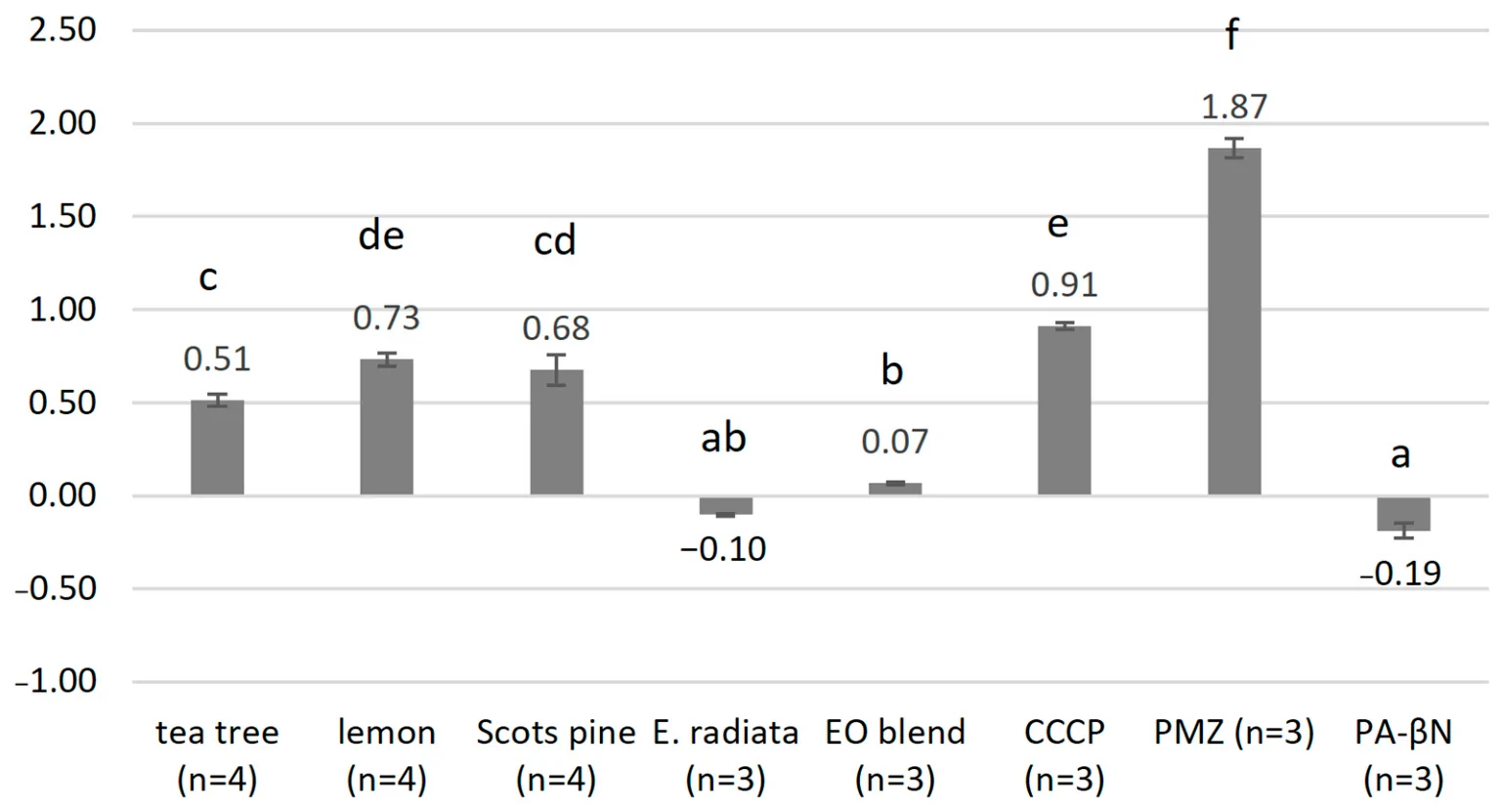
Relative fluorescence index (RFI) values of the EOs and controls in *E. coli* 130063. Bars represent mean RFI values, with error bars indicating the standard error of the mean (SEM); individual *n* values are indicated below the treatment labels. Different lowercase letters indicate statistically significant differences among treatments according to Tukey’s post hoc test (*p* < 0.05); treatments sharing at least one letter are not significantly different.

**Figure 3:**
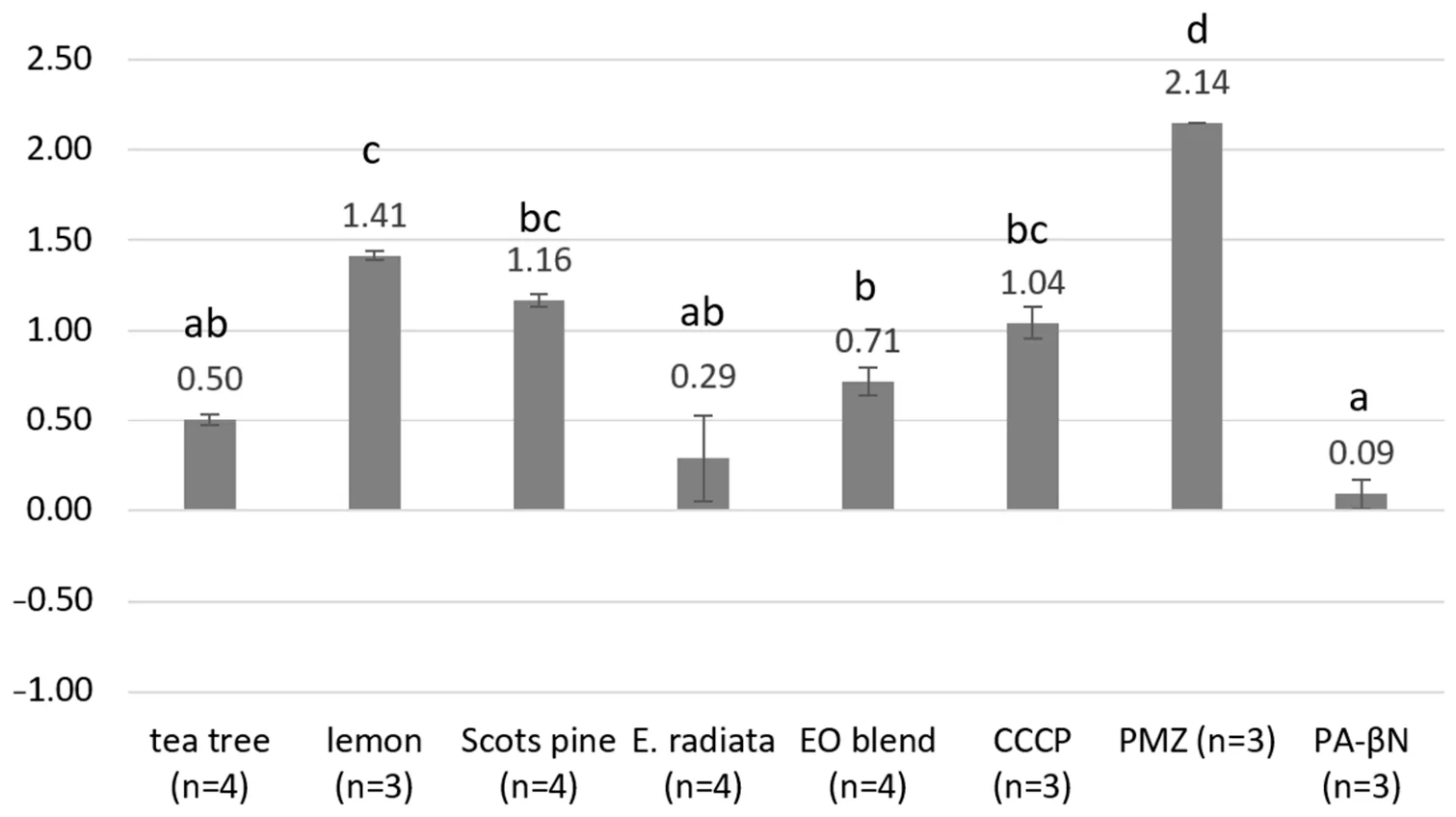
Relative fluorescence index (RFI) values of the EOs and controls in *K. pneumoniae* 33443. Bars represent mean RFI values, with error bars indicating the standard error of the mean (SEM); individual *n* values are indicated below the treatment labels. Different lowercase letters indicate statistically significant differences among treatments according to Tukey’s post hoc test (*p* < 0.05); treatments sharing at least one letter are not significantly different.

**Figure 4:**
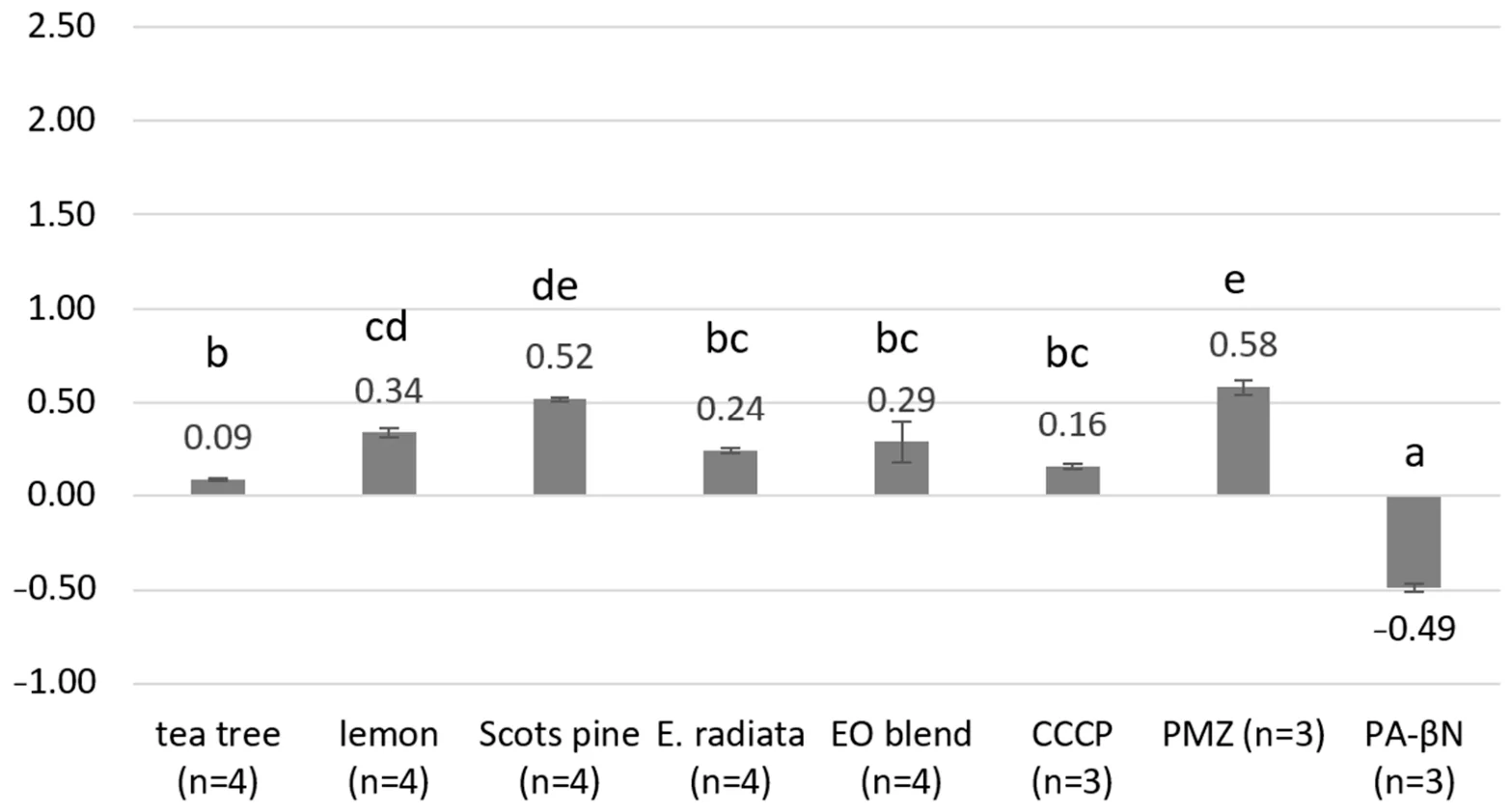
Relative fluorescence index (RFI) values of the EOs and controls in *K. pneumoniae* 33163. Bars represent mean RFI values, with error bars indicating the standard error of the mean (SEM); individual *n* values are indicated below the treatment labels. Different lowercase letters indicate statistically significant differences among treatments according to Tukey’s post hoc test (*p* < 0.05); treatments sharing at least one letter are not significantly different.

The time-course fluorescence profiles obtained during the 60 min EB accumulation assay are presented in Supplementary Figure S1 and illustrate the temporal changes in EB-associated fluorescence for the tested EOs and positive controls in each bacterial strain. Based on these measurements, relative fluorescence index (RFI) values were calculated to quantitatively compare intracellular EB accumulation, with higher positive RFI values indicating greater accumulation (Figure 1, Figure 2, Figure 3 and Figure 4). One-way ANOVA revealed an overall effect of treatment for each bacterial strain (*p* < 0.05). Detailed pairwise statistical differences (based on Tukey’s post hoc test) are indicated in Figure 1, Figure 2, Figure 3 and Figure 4.

For *E. coli* 128673, RFI values differed significantly among treatments (one-way ANOVA, *p* < 0.05; Figure 1). Tea tree EO and the EO blend produced relatively high positive RFI values that did not differ significantly from those obtained with CCCP and PMZ. In contrast, lemon, Scots pine, and *E. radiata* showed significantly lower RFI values than these treatments, while PA-βN produced a negative RFI value.

For *E. coli* 130063, RFI values also differed significantly among treatments (one-way ANOVA, *p* < 0.05; Figure 2). *E. radiata*, the EO blend, and PA-βN produced the lowest RFI values, with negative values observed for *E. radiata* and PA-βN. Among the EOs, lemon and Scots pine produced relatively higher positive RFI values. Lemon EO did not differ significantly from CCCP, whereas PMZ produced the highest RFI value and differed significantly from all other treatments.

For *K. pneumoniae* 33443, significant differences in RFI values were observed among treatments (one-way ANOVA, *p* < 0.05; Figure 3). Among the EOs, lemon and Scots pine produced the highest mean RFI values (1.41 and 1.16, respectively), although neither differed significantly from CCCP. Tea tree and *E. radiata* showed comparatively lower RFI values. PMZ produced the highest RFI value overall and was significantly different from all other treatments.

For *K. pneumoniae* 33163, RFI values differed significantly among treatments (one-way ANOVA, *p* < 0.05; Figure 4). PA-βN produced the lowest RFI value (−0.49). Among the EOs, Scots pine showed the highest mean RFI value (0.52), followed by lemon (0.34), the EO blend (0.29), *E. radiata* (0.24), and tea tree (0.09). PMZ produced the highest RFI value among the positive controls and did not differ significantly from Scots pine.

Among the known efflux pump inhibitors used as positive controls, PMZ consistently produced the highest RFI values (1.39–2.14), followed by CCCP (0.16–1.14), reflecting their effects on intracellular EB accumulation. PA-βN generally produced the lowest RFI values, including negative values in some strains, indicating fluorescence levels below those of the untreated control under the present assay conditions. Overall, *E. coli* 128673 and *K. pneumoniae* 33443 generally showed greater increases in RFI in response to the tested treatments, whereas *E. coli* 130063 and *K. pneumoniae* 33163 exhibited lower RFI responses. These findings further support the strain-dependent nature of EO-associated changes in EB accumulation.

## 3. Discussion

The increasing prevalence of multidrug-resistant (MDR) Gram-negative pathogens represents a major challenge in healthcare settings, highlighting the need for complementary strategies that may enhance the effectiveness of existing antimicrobial approaches. In the present study, selected commercially available EOs and their blend exhibited strain-dependent antibacterial effects against clinical isolates of MDR *E. coli* and *K. pneumoniae*. Among the tested microorganisms, *E. coli* strains showed greater susceptibility than *K. pneumoniae*, for which MIC values were 5 mg/mL or above. This difference is consistent with the intrinsic resistance characteristics of *K. pneumoniae*, including its highly restrictive outer membrane and efficient multidrug efflux systems, which together contribute to reduced susceptibility toward structurally diverse antimicrobial compounds [26]. Tea tree oil, *E. radiata* oil, and the EO blend showed measurable antibacterial effects against the *E. coli* strains, while *E. radiata* exhibited the lowest MIC value against *E. coli* 130063. It should be emphasized that most of the MIC values observed in the present study were relatively high compared with concentrations typically considered promising for further antimicrobial development. Only *E. radiata* EO against both *E. coli* strains (0.625 and 0.312 mg/mL) and the EO blend against *E. coli* 130063 (0.625 mg/mL) showed MIC values below 1 mg/mL. Therefore, particularly the MIC values of 2.5–5 mg/mL or higher should not be interpreted as indicating potential systemic therapeutic efficacy. Rather, the present antibacterial data should be considered as in vitro screening results supporting further investigation of selected EOs in complementary or application-specific antimicrobial strategies.

The EO blend warrants particular consideration because its biological effects cannot be attributed unequivocally to a single constituent or individual EO. GC-MS analysis showed that the blend was dominated by eucalyptol (37.71%), limonene (19.48%), *α*-pinene (11.68%), and *β*-pinene (5.38%). Nevertheless, its antibacterial and EB accumulation profiles did not simply reproduce those of any single constituent EO. For example, the blend produced its highest RFI value against *E. coli* 128673 (1.27), suggesting that interactions among its constituents may influence resistance-associated responses. However, because the individual oils were not experimentally recombined at defined ratios and interaction studies were not performed, additive or synergistic effects cannot be concluded from the present data and should be investigated in future studies.

In contrast, lemon oil showed limited antibacterial activity under the tested conditions. These findings indicate that the antibacterial effects of EOs depend on both bacterial background and chemical composition.

Beyond direct antibacterial effects, increasing attention has been directed toward the potential ability of natural compounds to modulate bacterial resistance mechanisms, including multidrug efflux systems. In Enterobacterales, the AcrAB-TolC resistance-nodulation-division (RND) efflux system represents one of the most important contributors to multidrug resistance by actively exporting a broad range of antimicrobial compounds from bacterial cells [27]. Therefore, compounds capable of interfering with efflux-associated processes may increase intracellular accumulation of antimicrobial molecules and contribute to resistance modulation [27]. In the present study, the EB accumulation assay revealed strain-dependent increases in intracellular fluorescence following EO exposure. Tea tree and Scots pine EOs produced moderate increases in intracellular EB accumulation across multiple strains. An interesting apparent dissociation was observed for lemon EO, which showed little direct antibacterial effect within the tested concentration range (MIC >5 mg/mL) but produced the highest EO-associated RFI value in *K. pneumoniae* 33443 (1.41). This finding suggests that growth inhibition and increased intracellular EB accumulation represent distinct biological endpoints and are not necessarily directly correlated. Moreover, increased EB fluorescence may reflect not only reduced efflux but also EO-induced changes in membrane permeability, membrane organization, or membrane-associated energy gradients. Therefore, the pronounced RFI response to lemon EO should not be interpreted as evidence of potent direct efflux pump inhibition, but rather as an indication that the oil influences one or more processes governing intracellular EB accumulation.

The EO blend also produced a moderate positive RFI response, with the highest RFI observed against *E. coli* 128673 (RFI = 1.27). However, these results should be interpreted cautiously, as increased EB accumulation does not exclusively indicate direct efflux pump inhibition. Alterations in membrane permeability, membrane fluidity, or proton motive force may also influence intracellular dye accumulation, particularly because many bacterial efflux systems, including RND transporters, depend on membrane-associated energy gradients [28]. Because RND transporters such as AcrAB-TolC are driven by the proton motive force, membrane-active EO constituents may indirectly reduce efflux by dissipating the transmembrane electrochemical gradient without directly interacting with or inhibiting the AcrB transporter.

The observed strain-dependent differences in EO-mediated effects on EB accumulation may reflect differences in bacterial resistance backgrounds, outer membrane properties, or the expression of resistance-associated determinants, although these mechanisms were not directly investigated in the present study. Notably, *E. coli* 128673 and *K. pneumoniae* 33443 showed higher responsiveness toward both EO treatments and positive controls, whereas *E. coli* 130063 and *K. pneumoniae* 33163 exhibited lower RFI values. These findings suggest that bacterial response to EOs may vary according to strain-specific characteristics. Overall, no simple relationship was evident between antibacterial potency and EO-induced EB accumulation. *E. radiata* showed the strongest direct antibacterial effect against *E. coli* 130063 but produced little or no increase in EB accumulation in this strain, whereas lemon EO showed weak direct antibacterial activity but a pronounced RFI response in *K. pneumoniae* 33443. These findings indicate that direct growth inhibition and modulation of processes affecting intracellular EB accumulation are at least partly independent phenomena. Furthermore, for EO-strain combinations with MIC >5 mg/mL, the 2.5 mg/mL concentration used in the EB accumulation assay was not a confirmed sub-MIC concentration. Consequently, RFI values obtained under these conditions, particularly the response of lemon EO in *K. pneumoniae* 33443, should be interpreted cautiously. The mechanism underlying negative RFI values could not be resolved in the present study and requires dedicated membrane-integrity and fluorescence-interference controls.

Based on previous studies, the physicochemical characteristics of the major EO constituents may contribute to the antibacterial and efflux-associated effects observed in the present study. The distinct chemical profiles of the investigated EOs may help to interpret, at least in part, the different antibacterial and EB accumulation responses observed in the present study. Tea tree EO, characterized predominantly by terpinen-4-ol, showed measurable antibacterial effects against both *E. coli* strains and also produced increased EB accumulation, particularly in *E. coli* 128673. The membrane-interacting properties previously described for terpinen-4-ol may therefore be compatible with both growth inhibition and changes in processes governing intracellular EB accumulation [29]. Scots pine EO, rich in *α*-pinene, showed comparatively weak direct antibacterial effects but produced moderate increases in RFI in several strains, suggesting that its influence on membrane-associated processes may occur even in the absence of pronounced growth inhibition [30]. A particularly informative result was obtained with limonene-rich lemon EO, which showed little direct antibacterial effect within the tested concentration range but produced a high RFI in *K. pneumoniae* 33443. This dissociation supports the view that membrane-related effects influencing EB accumulation do not necessarily translate into direct growth inhibition [31]. Conversely, eucalyptol-rich *E. radiata* EO exhibited the lowest MIC against *E. coli* 130063 but did not increase EB accumulation in this strain, indicating that its antibacterial effect is unlikely to depend primarily on modulation of EB efflux-associated processes and may involve other cellular mechanisms [32]. Taken together, these findings suggest that neither antibacterial potency nor EB accumulation can be predicted solely from the predominant EO constituent, and that the overall chemical matrix, minor constituents, strain-specific properties, and the biological endpoint examined are likely to influence the observed responses. Previous studies suggest that the biological effects of EOs may result from interactions among multiple constituents, with additive or synergistic interactions between major and minor components potentially contributing to their overall antimicrobial effects [33]. However, such interactions were not directly assessed in the present study.

When compared with previous reports, our findings further emphasize that the antibacterial effect of an EO cannot be predicted solely from the abundance of its major constituent. For example, the relatively strong antibacterial effect of *E. radiata* EO against *E. coli* 130063 occurred despite eucalyptol being its predominant constituent, suggesting that the overall EO matrix and interactions among constituents may substantially influence biological effects. Conversely, the limonene-rich lemon EO showed little direct growth inhibition within the tested concentration range, despite the previously reported membrane-disruptive properties of limonene. These observations support the concept that EO effects are determined not only by individual major compounds but also by their relative proportions, minor constituents, bacterial strain characteristics, and the biological endpoint examined.

From a clinical perspective, the focus of the present study on MDR Gram-negative Enterobacterales is justified by their recognized importance among antimicrobial resistance priority pathogens and their significant contribution to healthcare-associated infections [34]. The clinical isolates investigated in this study were selected based on their relevance as patient-derived MDR *E. coli* and *K. pneumoniae* strains available from our hospital setting, representing clinically important resistance phenotypes encountered in Hungary. Nevertheless, the limited number of isolates represents one of the limitations of the study, and future investigations including larger collections of geographically diverse MDR strains would be necessary to confirm the broader applicability of these findings.

Additional limitations include the in vitro nature of the experiments and the indirect character of the EB accumulation assay. Furthermore, the intrinsic fluorescence and potential quenching effects of the EOs were not separately assessed; therefore, fluorescence interference cannot be completely excluded. Future studies should include appropriate fluorescence interference controls to determine whether the observed changes in EB fluorescence reflect actual changes in intracellular EB accumulation and to further clarify their relationship to efflux-associated processes.

Although increased fluorescence is frequently interpreted as reduced efflux-mediated transport, additional mechanisms such as membrane permeabilization or partial membrane depolarization may contribute to the observed effects. Therefore, future studies should include molecular approaches such as expression analysis of efflux-related genes (acrA, acrB, tolC), transporter-specific assays, and membrane integrity measurements to further clarify the underlying mechanisms.

Taken together, the present findings suggest that tea tree, Scots pine, *E. radiata*, and lemon EOs, as well as the EO blend, may influence resistance-associated bacterial responses in a strain-dependent manner. Rather than serving as direct replacements for conventional antimicrobials, these EOs warrant further mechanistic investigation to determine whether their effects on resistance-associated processes may have potential complementary antimicrobial relevance.

## 4. Materials and Methods

### 4.1. GC-MS

The EOs used in this study were obtained commercially from Herby’s Ltd. (Pécs, Hungary). Commercially available EOs were selected to investigate products representative of materials that could realistically be considered for future practical applications. To ensure analytical traceability despite their commercial origin, individual LOT numbers were recorded and the chemical composition of each EO was independently characterized by GC-MS. The EOs included in this study were tea tree (*Melaleuca alternifolia*; LOT number: TFB006), lemon (*Citrus limon*; LOT number: CIB006), Scots pine (*Pinus sylvestris*; LOT number: EFO009), *Eucalyptus radiata* (LOT number: ERB001), and their blend. The EO blend, containing a mixture of the aforementioned oils, is commercially available (Herby’s Ltd., Hungary). The exact formulation of the EO blend is proprietary and therefore cannot be disclosed in accordance with the agreement with the manufacturer. It contains a higher proportion of tea tree and *E. radiata* oils. Because the exact proportions of the individual EOs in the commercial blend are proprietary, independent reconstruction of the formulation is not possible. This represents a limitation of the present study. Nevertheless, the analyzed blend is traceable as a commercial product, and its experimentally determined GC-MS profile is provided to enable chemical comparison with future batches or related formulations. Prior to analysis, the EOs were stored at 4 °C in tightly closed amber glass bottles and protected from light. GC-MS characterization was performed immediately after opening the bottles. The same LOTs characterized by GC-MS were subsequently used for all microbiological experiments.

Dichloromethane (DCM) and saturated C7–C30 n-alkane homologous series were purchased from Merck KGaA (Darmstadt, Germany). A solvent blank (DCM), the homologous series, and EO sample solutions diluted 1:1000 and 1:10,000 in DCM were analyzed using an Agilent 7890B gas chromatograph (Agilent Technologies, Santa Clara, CA, USA) coupled to an Agilent 7010B mass spectrometer (Agilent Technologies, Santa Clara, CA, USA). Measurements were conducted in MS1 scan mode with electron ionization (EI). Separation of the compounds was achieved on an HP-5ms capillary column (5% diphenyl/95% dimethylpolysiloxane; 30 m × 0.25 mm i.d. × 0.25 µm film thickness; Agilent Technologies, Santa Clara, CA, USA). The GC system was fitted with a split/splitless injector, with the injector maintained at 280 °C. Helium (5.0) (Messer Hungarogáz Kft., Budapest, Hungary) served as the carrier gas at a constant linear velocity of 30.0 cm/s. A linear oven temperature program was applied from 50 °C to 350 °C at a heating rate of 3 °C/min, with a final hold of 5 min at 350 °C. The interface and ion source temperatures were set to 200 °C and 250 °C, respectively. Data acquisition was performed over an m/z range of 40–550. All analyses were carried out using triplicate injections; thus, the resulting area percentages were reported to one decimal place. Semi-quantitative results were calculated from the relative peak area percentages, without applying any additional correction factors. EO constituents were identified in GC-MS analyses predominantly using the FFNSC 4.0 library (Shimadzu, Kyoto, Japan), with the Hochmuth GC-MS library used as a supplementary reference [35]. The compound names were assigned according to the nomenclature used in the corresponding mass spectral library. For each detected compound, the experimental linear retention index (LRI_exp_) was calculated using a C7–C30 n-alkane homologous series analyzed under the same temperature-programmed chromatographic conditions. The resulting LRI_exp_ values were compared with library linear retention indices (LRI_lib_), as reported in Table S1. Compound identification was based on combined mass spectral and retention-index matching, with a mass spectral similarity score > 90% and an LRI deviation within ±5 index units.

### 4.2. Bacterial Strains

The study focused on clinically relevant MDR pathogens associated with healthcare-associated infections and possessing well-characterized efflux systems. The antibacterial activity of the EO samples was evaluated against the Gram-negative bacteria *Escherichia coli* and *Klebsiella pneumoniae*. Two strains per species were tested, representing different antimicrobial resistance profiles. Clinical strains of *K. pneumoniae* 33443 and *K. pneumoniae* 33163 [13] and *E. coli* 128673 and *E. coli* 130063 [36] were provided by the Department of Medical Microbiology at the University of Szeged and were included in the study (ethics approval case number: BM/27890-3/2024). The species identities of the clinical isolates were confirmed by both MALDI-TOF MS and conventional biochemical methods. The antibiotic-resistance data of *K. pneumoniae* strains were previously published [13]. Both *E. coli* strains were clinical isolates from midstream urine samples and were ESBL-producing isolates. The antibiotic-resistance data of the *E. coli* strains can be found in a previous study [36]. These species are well-recognized HAI pathogens associated with substantial morbidity and antimicrobial resistance. All bacterial isolates were suspended in Mueller–Hinton Broth (MHB, Biolab Ltd., Budapest, Hungary). Starter cultures were prepared from fresh colonies, and bacterial suspensions were incubated to obtain actively growing cells for the experiment. The exact resistance profile of the bacteria is shown in Table 3. Antimicrobial susceptibility testing was performed using the disk diffusion method. Inhibition zone diameters were interpreted as susceptible (S), susceptible, increased exposure (I), or resistant (R) according to the EUCAST Clinical Breakpoint Tables applicable at the time of testing: version 11.0 (2021) for the *K. pneumoniae* isolates and version 16.0 (2026) for the *E. coli* isolates [37].

### 4.3. Minimum Inhibitory Concentrations

The minimum inhibitory concentrations (MICs) of the EO samples were determined using the broth microdilution method in 96-well microtiter plates, following the guidelines of the Clinical and Laboratory Standards Institute [38]. MHB was used as the growth medium for the bacteria. Bacterial suspensions were prepared at a concentration of 10^5^ CFU/mL. The bacterial inoculum was standardized based on optical density. Briefly, bacterial suspensions were adjusted to an OD_600_ of 0.6 using a spectrophotometer (BMG Labtech SPECTROstar Nano, Budapest, Hungary) and subsequently diluted (100×) in MHB to obtain a final inoculum concentration of approximately 1 × 10^5^ CFU/mL. The relationship between the optical density and viable bacterial concentration was established by viable colony counting. This standardized procedure was applied consistently to all investigated isolates to ensure comparable initial bacterial densities.

The EOs were tested at concentrations ranging from 5.000 to 0.002 mg/mL using two-fold serial dilutions. Tween 40 (Sigma-Aldrich Kft., Budapest, Hungary) was used as a solubilizing agent. Tween 40 was included as an emulsifier control at a concentration of 0.5% in the stock solution and showed no inhibitory effect. The MIC was defined as the lowest concentration at which no visible bacterial growth was observed after 18 h of incubation at 37 °C. Antibiotic controls were used according to the sensitivity tests (gentamicin for *E*. *coli* 128673 and meropenem for *K. pneumoniae* 33163, *K. pneumoniae* 33443, and *E. coli* 130063). MIC determinations were performed in four independent replicates, consistent with the methodology used in our previous study [39].

### 4.4. Ethidium Bromide Accumulation Assay

The impact of EOs on EB accumulation was assessed using an automated EB assay on a CLARIOstar Plus plate reader (BMG Labtech, Aylesbury, UK). Bacterial cultures were grown to mid-logarithmic phase (OD_600_ = 0.6), after which the cells were collected, washed three times in phosphate-buffered saline (PBS; pH 7.4), centrifuged at 13,000× *g* for 3 min, and resuspended in fresh PBS. The EOs were generally tested at half their respective MIC (MIC/2), which was selected as a sub-inhibitory concentration to minimize interference from direct growth inhibition during the EB accumulation assay. For EO–bacterium combinations in which the MIC exceeded the highest concentration tested (>5.0 mg/mL), 2.5 mg/mL was used as an operational test concentration corresponding to half of the highest concentration examined, rather than a confirmed MIC/2. All samples were mixed with PBS containing EB at a concentration of 2 μg/mL. For the assay, 50 μL of the EB-EO mixture was dispensed into each well of a black 96-well microtiter plate (Greiner Bio-One Hungary Kft., Mosonmagyaróvár, Hungary), followed by 50 μL of the bacterial suspension adjusted to OD_600_ = 0.6. Carbonyl cyanide m-chlorophenyl hydrazone (CCCP, Merck Life Sciences, Budapest, Hungary; 250 μM), promethazine (PMZ, Merck Life Sciences, Budapest, Hungary; 200 μg/mL), and phenylalanine-arginine β-naphthylamide (PA-βN, Merck Life Sciences, Budapest, Hungary; 100 μg/mL) were used as the positive controls. Fluorescence was recorded in real time for 60 min using a CLARIOstar plate reader, with excitation and emission wavelengths set to 525 and 615 nm, respectively. Measurements were performed in three or four independent replicates, as indicated in Figure 1, Figure 2, Figure 3 and Figure 4. Based on the fluorescence kinetics, the effect of each sample on EB accumulation was expressed as the relative fluorescence index (RFI), calculated at the final time point (60 min) using the following equation:

RFI = (RF_treated − RF_untreated)/RF_untreated

where RF_treated represents the fluorescence at the last time point in the presence of the test sample, and RF_untreated corresponds to the fluorescence of the solvent control (Tween 40). A vehicle control containing Tween 40 at the same final concentration as in the EO-treated samples was included throughout the 60 min fluorescence assay to account for any effect of the surfactant on EB accumulation or membrane properties. Positive RFI values indicate higher fluorescence in treated cells relative to the untreated control and are consistent with increased intracellular EB accumulation, whereas values close to zero indicate little or no detectable change. Negative RFI values indicate lower fluorescence than in the untreated control and therefore do not provide evidence of increased EB accumulation or efflux inhibition. Such values should not be interpreted as direct evidence of enhanced efflux, as other factors affecting EB uptake, membrane properties, or fluorescence may also contribute [40]. EO-specific autofluorescence and fluorescence quenching were not independently quantified under the assay conditions, which represents a limitation of the present study.

### 4.5. Statistical Analysis

RFI data are presented as mean ± standard error of the mean (SEM) based on three or four independent measurements; the number of measurements is indicated by *n* in the figures. Differences among treatments were analyzed separately for each bacterial strain using one-way analysis of variance (ANOVA), followed by Tukey’s multiple-comparison test. Differences were considered statistically significant at *p* < 0.05. Statistical analyses were performed using SPSS Statistics for Windows, version 20.0. MIC values were derived from a two-fold serial dilution assay and were therefore analyzed descriptively rather than by parametric statistical testing.

## 5. Conclusions

The present study demonstrated strain-dependent in vitro antibacterial effects of the selected EOs against MDR *E. coli* and *K. pneumoniae*, with substantially greater susceptibility observed for *E. coli*. Among the tested samples, *E. radiata* showed the lowest MIC values against *E. coli* (0.312–0.625 mg/mL), while the EO blend also showed an MIC below 1 mg/mL against *E. coli* 130063. In contrast, no EO inhibited the *K. pneumoniae* isolates at concentrations below 5 mg/mL, indicating comparatively low antibacterial potency against these strains. These MIC values should not be interpreted as being comparable to the antibacterial potency of conventional antibiotics or as evidence of systemic therapeutic efficacy. Importantly, the study extends previous knowledge on the antibacterial properties of these EOs by evaluating chemically characterized, commercially available oils and their blend against MDR clinical isolates and by examining antibacterial effects together with changes in intracellular EB accumulation. The lack of a consistent relationship between MIC and RFI values indicates that antibacterial potency and EB accumulation represent distinct biological responses and that changes in EB accumulation cannot be attributed unequivocally to direct efflux pump inhibition. Thus, the present findings provide a basis for distinguishing direct antibacterial effects from potential modulation of resistance-associated processes in future studies. The natural origin and commercial availability of EOs may offer practical advantages for the development of complementary antimicrobial approaches; however, their cost-effectiveness, safety, formulation requirements, and acceptance in specific healthcare applications remain to be established. Future studies should specifically investigate the underlying mechanisms using dedicated efflux and membrane-function assays, evaluate EO–antibiotic interactions using appropriate combination methods, and assess the most promising EOs in application-specific models before their potential use in non-systemic or environmental antimicrobial settings can be established.

## Figures and Tables

**Table 1:** Minimum Inhibitory Concentrations (MICs) of the EOs and antibiotics (mg/mL) used in this study.

Test Sample	*E. coli* 128673	*E. coli* 130063	*K. pneumoniae* 33443	*K. pneumoniae* 33163
tea tree	1.250	1.250	>5.000	>5.000
lemon	>5.000	>5.000	>5.000	>5.000
Scots pine	5.000	2.500	>5.000	>5.000
*E. radiata*	0.625	0.312	5.000	>5.000
EO blend	1.250	0.625	5.000	>5.000
gentamicin	3.125 × 10^−3^	7 × 10^−6^	7 × 10^−6^	7 × 10^−6^
meropenem	7 × 10^−6^	7 × 10^−6^	7 × 10^−6^	7 × 10^−6^

**Table 2:** EO concentrations used in ethidium bromide accumulation assay (mg/mL).

Test Sample	*E. coli* 128673	*E. coli* 130063	*K. pneumoniae* 33443	*K. pneumoniae* 33163
tea tree	0.625	0.625	2.500	2.500
lemon	2.500	2.500	2.500	2.500
Scots pine	2.500	1.250	2.500	2.500
*E. radiata*	0.312	0.156	2.500	2.500
EO blend	0.625	0.312	2.500	2.500

**Table 3:** Antibiotic resistance profile of bacteria included in this study and examined with disk-diffusion assay (S: susceptible, R: resistant, I: susceptible, increased exposure, ESBL: extended-spectrum beta-lactamase producing strain).

	*K. pneumoniae* 33443 [13]	*K. pneumoniae* 33163 [13]	*E. coli* 128673 [36]	*E. coli* 130063 [36]
Ampicillin	R	R	R	R
AMC (amoxicillin, clavulic acid)	S	R	R	R
Cefuroxime	S	R	R	R
Ceftriaxone	S	R	R	R
Ceftazidime	S	R	R	R
Ceftazidime/Avibactam	S	S	R	R
Cefixime			R	R
Ciprofloxacin	R	R	S	R
TMP/SMX (Sumetrolim)	S	R	R	R
Ertapenem	S	S	S	S
Imipenem	S	S	S	S
Meropenem	S	S	S	S
Gentamicin	S	R	S	R
Tobramycin	S	I		
Amikacin	S	I		
Norfloxacin	R	R	S	R
Nitrofurantoin			S	R
Fosfomycin			S	S
ESBL			YES	YES

## Data Availability

The data supporting the findings of this study are available within the article and its Supplementary Materials. Additional data are available from the corresponding author upon reasonable request.

## Supplementary Materials

The following supporting information can be downloaded at: https://www.mdpi.com/article/10.3390/plants15172587/s1, Table S1: Semi-quantitative volatile composition of Scots pine (SPEO), tea tree (TTEO), *Eucalyptus radiata* (EREO), lemon (LEO) essential oils and their blend; Figure S1: Time-course ethidium bromide (EB) fluorescence profiles of the tested essential oils and positive controls in *E. coli* 128673, *E. coli* 130063, *K. pneumoniae* 33443, and *K. pneumoniae* 33163 during the 60-min accumulation assay. Fluorescence is expressed in arbitrary units.

## Abbreviations

The following abbreviations are used in this manuscript:

AMR	Antimicrobial resistance
CCCP	Carbonyl cyanide 3-chlorophenylhydrazone
EB	Ethidium bromide
EO	Essential oil
EREO	*Eucalyptus radiata* essential oil
HAI	Hospital-acquired infection
LEO	Lemon essential oil
MDR	Multidrug-resistant
MHB	Mueller-Hinton Broth
PA-βN	Phenylalanine-arginine β-naphthylamide
PBS	Phosphate-buffered saline
PMZ	Promethazine
RFI	Relative fluorescence index
SPEO	Scots pine essential oil
TTEO	Tea tree essential oil
