# Supplementary material for: Selected Essential Oils Modulate Ethidium Bromide Accumulation and Exhibit Antibacterial Activity Against Multidrug-Resistant Escherichia coli and Klebsiella pneumoniae
Source: Plants (Basel). 2026 Aug 25;15(17):2587. doi: 10.3390/plants15172587 (PMC13567068; doi:10.3390/plants15172587)
Supplement: Supplementary file 1 [file plants-15-02587-s001.zip › plants-4480271-supplementary.pdf]

Table S1. Semi-quantitative volatile composition of Scots pine (SPEO), tea tree (TTEO), *E. radiata* (EREO), lemon (LEO) essential oils and their blend.

**Compound**	**LRI_{lib}**	**LRI_{exp}**	**Area percentages (%)**				
			**SPEO**	**TTEO**	**EREO**	**LEO**	**EO blend**
Hexan-2-ol	802	807	tr	tr	tr	tr	tr
Hex-(2E)-enal	850	855	-	-	tr	-	-
Santene	880	884	0.1	-	-	-	0.1
Furan <2-butyl->	890	895	-	-	-	tr	-
Santolinatriene	902	905	tr	-	-	-	tr
Tricyclene	923	921	0.2	-	-	-	0.1
Thujene <*alpha*->	927	926	tr	1.1	0.4	0.6	0.4
Pinene <*alpha*->	933	932	38.7	3.0	2.8	2.3	11.7
Fenchene <*alpha*->	950	945	-	tr	tr	-	-
Camphene	953	948	1.9	tr	tr	0.1	1.1
Thuja-2,4(10) -diene	953	952	0.1	-	-	-	tr
Cymene <*meta*->	976	971	0.9	-	-	-	tr
Sabinene	972	972	tr	tr	1.4	2.1	0.4
Pinene <*beta*->	978	975	15.2	0.8	0.8	13.7	5.4
Menth-3-ene <*para*->	986	983	-	tr	-	-	-
Mentha-2,8-diene <*trans*-, *meta*->	982	983	0.1	tr	-	-	tr
Pinane <*cis*->	988	989	0.1	-	-	-	tr
Myrcene	991	991	2.6	0.4	1.1	1.4	0.8
Carene <*delta*-2->	1000	1001	tr	-	-	-	-
Mentha-1(7),8-diene <*meta*->	1001	1003	0.2	-	-	-	-
Mentha-1(7),8-diene <*para*->	1004	1004	0.3	-	-	-	-
Phellandrene <*alpha*->	1007	1005	0.1	tr	0.4	tr	0.1
Carene <*delta*-3->	1009	1010	11.3	-	-	tr	3.2
Cineole <1,4->	1017	1014	0.1	tr	-	-	-
Terpinene <*alpha*->	1018	1016	-	0.1	0.1	0.2	0.8
Cymene <*ortho*->	1024	1021	0.1	-	-	-	-
Menth-1-ene <*para*->	1023	1021	tr	tr	-	-	tr
Cymene <*para*->	1025	1024	2.7	26.9	1.2	1.4	4.7
Phellandrene <*beta*->	1031	1028	-	0.6	-	-	-
Limonene	1030	1029	8.5	0.9	2.7	64.8	19.5
Eucalyptol	1032	1030	0.3	6.0	75.0	0.2	37.7
Ocimene <(*Z*)-, *beta*->	1035	1038	0.1	tr	tr	tr	tr
Ocimene <(*E*)-, *beta*->	1049	1046	-	tr	0.1	0.1	tr
Terpinene <*gamma*->	1058	1058	-	2.4	0.4	10.2	3.7
Sabinene hydrate <*cis*->	1069	1067	-	-	tr	tr	-
Mentha-3,8-diene <*para*->	1073	1070	tr	tr	-	-	tr
Mentha-3,8-diene <*para*->	1073	1071	tr	-	-	-	-
Linalool oxide <*cis*->	1069	1072	-	-	tr	-	-

Rose oxide <*trans*-dihydro->	1073	1076	-	0.1	-	-	-
Terpinolene	1086	1088	-	0.6	0.1	0.4	0.5
Fenchone	1090	1088	tr	-	-	-	-
Cymenene <*para*->	1093	1091	0.1	0.1	-	-	tr
Epoxymyrcene <6,7->	1096	1094	tr	-	-	-	-
Pinene oxide <*alpha*->	1101	1098	0.4	-	-	-	-
Sabinene hydrate <*trans*->	1099	1099	-	tr	tr	tr	tr
Linalool	1101	1101	-	tr	0.2	tr	tr
Menthatriene <1,3,8-*para*-> Isomer III	1106	1102	0.1	-	-	-	-
Menthatriene <1,3,8-*para*-> Isomer I	1106	1106	0.3	-	-	-	tr
Nonanal <*n*->	1107	1107	-	-	-	tr	-
Menthatriene <1,3,8-*para*-> Isomer II	1106	1111	0.8	tr	-	-	-
Fenchol <endo->	1119	1114	0.1	-	-	-	tr
Chrysanthenone	1122	1117	0.1	-	-	-	-
Mentha-2,8-dien-1-ol <*trans-, para*->	1122	1121	0.1	-	-	-	-
Menth-2-en-1-ol <*cis-, para*->	1124	1122	-	0.1	0.2	-	tr
Campholenal <*alpha*->	1125	1127	tr	-	-	-	tr
Ocim-(4E,6Z)-ene <*allo*->	1128	1130	-	-	-	tr	-
Limonene oxide <*cis*->	1134	1133	tr	-	-	tr	-
Mentha-2,8-dien-1-ol <*cis-, para*->	1138	1135	0.1	0.1	tr	-	tr
Verbenol <*cis*->	1141	1136	-	tr	-	-	-
Pinocarveol <*trans*->	1141	1138	0.7	-	-	-	-
Limonene oxide <*trans*->	1138	1138	-	-	-	tr	-
Menth-2-en-1ol <*trans-para*->	1139	1140	-	tr	0.1	-	tr
Sabinol <*trans*->	1140	1141	0.1	-	-	-	tr
Verbenol <*trans*->	1145	1144	0.5	-	-	-	-
Camphor	1149	1144	-	-	-	tr	tr
Eucarvone	1150	1152	tr	-	-	-	-
Citronellal	1152	1155	-	-	-	tr	tr
Chrysanthemol <*trans*->	1159	1160	-	tr	-	-	-
Pinocamphone <*trans*->	1160	1161	tr	-	-	-	-
Pinocarvone	1164	1162	0.6	-	-	-	tr
Thujol	1169	1165	0.2	-	-	-	-
Borneol	1169[a]	1165	-	-	-	-	tr
Chrysanthemol <*cis*->	1166	1166	-	tr	-	tr	-
Terpineol <*delta*->	1170	1167	-	-	0.1	-	tr
Oxygenated monoterpene 1	-	1176	0.5	-	-	-	-
Terpinen-4-ol	1184	1178	-	38.6	1.6	tr	4.8
Isogeranial	1179	1185	-	-	-	tr	-
Pinocarveol <*cis*-> +<br>Cymen-8-ol <*para*->	1186	1186	0.3	-	-	-	-
Cymen-8-ol <*para*->	1189	1188	-	0.6	-	-	tr
Cryptone	1187	1188	0.1	-	-	-	tr
Terpineol <*alpha*->	1195	1192	0.7	4.0	8.1	0.1	1.5

Terpineol <*gamma-*>	1200	1195	-	-	0.1	-	0.1
Myrtenal	1197	1196	0.9	-	-	-	-
Dihydrocarveol <*neo*->	1198	1197	-	-	-	tr	-
Dihydrocarveol <1,6->	1203	1200	-	-	-	tr	-
2-Propionaldehyde <tolyl-, *para-*>	1205	1206	0.5	-	-	-	-
Decanal <*n-*>	1208	1208	-	-	-	tr	-
Oxygenated monoterpene 2	-	1209	-	-	tr	-	-
Verbenone	1208	1209	0.5	-	-	-	-
Piperitol <*trans-*>	1209	1209	-	tr	-	-	tr
Sabinene hydrate acetate <*cis-*>	1221	1218	-	-	-	-	tr
Carveol <*trans-*>	1223	1221	tr	-	-	-	-
Isophorone <4-methylene->	1223	1223	0.2	-	-	-	-
Mentha-1(7),8-dien-2-ol <*cis-*, *para-*>	1230	1225	-	-	-	tr	-
Piperiton epoxid	1232[a]	1227	-	0.1	-	-	tr
Citronellol	1232	1231	-	-	tr	-	-
Carveol <*cis-*>	1232	1233	tr	-	-	-	-
Dihydrocarveol <neoiso->	1230	1235	-	-	-	tr	-
Neral	1238	1243	-	-	tr	0.2	tr
Carvone	1246	1247	0.1	-	-	-	tr
Carvotanacetone	1249	1249	tr	-	-	-	-
Car-3-en-2-one	1249	1253	0.1	-	-	-	-
Pinocamphone <*trans*-2-hydroxy->	1253	1253	-	1.7	-	-	tr
Geraniol	1255	1256	-	-	0.2	-	-
Piperitone	1267	1257	-	-	0.1	-	tr
Chrysanthemumate <ethyl-, *trans-*>	1260[a]	1262	tr	-	-	-	-
Menth-2-en-1,4-diol	1269[a]	1271	tr	1.3	tr	-	tr
Geranial	1268	1274	-	-	0.1	0.4	tr
Verbenyl acetate <*cis-*>	1278	1276	tr	0.8	-	-	-
Phellandral	1277	1277	-	-	-	tr	-
Bornyl acetate	1285	1285	3.2	tr	-	-	1.2
Verbenyl acetate <*trans-*>	1289	1294	tr	-	-	-	tr
Menthyl acetate	1290	1295	1.0	-	-	-	-
Oxygenated monoterpene 3	-	1301	-	0.2	-	-	-
Carvacrol	1300	1305	-	0.1	-	-	-
Cryptone <4-hydroxy->	1318	1321	-	0.4	-	-	-
cis-Dihydrocarvone epoxide	1333	1328	-	0.1	-	-	-
Elemene <*delta-*>	1335	1337	-	-	tr	-	tr
Cubebene <*alpha-*>	1347	1349	-	0.1	-	-	-
Longipinene <*alpha-*>	1352	1349	0.2	-	-	-	-
Terpinyl acetate <*alpha-*>	1349	1350	-	-	1.9	-	0.3
Citronellyl acetate	1350	1355	-	-	-	tr	-
Mentholactone <*cis-*>	1364	1359	-	tr	-	-	-
Carvomenthyl acetate isomer	-	1364	-	0.1	-	-	-
Geranyl acetate <*cis-*>	1361	1366	-	-	-	0.3	tr

Cyclosativene	1367	1367	tr	-	-	-	tr
Longicyclene	1372	1369	tr	-	-	-	-
Isoledene	1372	1372	-	0.1	-	-	tr
Copaene <*alpha*->	1375	1376	0.1	0.2	-	tr	tr
Patchoulene <*beta*->	1383[a]	1378	-	0.1	-	-	tr
Geranyl acetate <*trans*->	1380	1385	-	-	-	0.1	tr
Sativene	1394	1389	tr	0.3	-	-	-
Elemene <*beta*->	1390	1393	-	-	tr	-	tr
Gurjunene <*alpha*->	1406	1399	-	0.4	tr	-	-
Tetradecane <*n*->	1400	1400	-	-	-	tr	-
Gurjunene <*alpha*->	1406	1409	-	-	tr	-	0.1
Longifolene	1412	1412	1.4	tr	-	-	0.1
Bergamotene <*alpha*-, *cis*->	1416	1415	-	-	-	tr	-
Caryophyllene <(*E*)->	1424	1419	0.7	0.2	0.1	0.2	0.2
Ylangene <*beta*->	1428	1423	tr	-	-	-	-
Maaliene <*gamma*->	1430	1428	-	0.1	-	-	tr
Calarene	1434	1431	-	tr	-	-	tr
Maaliene <*alpha*->	1438	1435	-	0.1	-	-	tr
Bergamotene <*alpha*-, *trans*->	1432	1436	-	-	-	0.4	tr
Aromadendrene	1438	1440	-	1.8	tr	-	0.2
Selina-5,11-diene	1445	1443	-	0.2	-	-	tr
Himachalene <*alpha*->	1449	1449	tr	-	-	-	-
Cadina-3,5-diene	1452	1450	-	-	-	-	tr
Humulene <*alpha*->	1454[a]	1454	0.1	tr	tr	tr	tr
Alloaromadendrene	1460	1456	-	0.7	-	-	0.1
Sesquisabinene	1455	1458	-	-	-	tr	-
Santalene <*beta*->	1459	1460	-	-	-	tr	-
Caryophyllene <9-epi-(*E*)->	1464	1461	-	-	tr	-	-
Cadina-1(6),4-diene	1475	1474	-	-	-	-	tr
Gurjunene <*gamma*->	1476	1477	-	0.1	-	-	-
Muurolene <*gamma*->	1478[a]	1478	tr	0.1	-	-	tr
Curcumene <*gamma*->	1482	1481	-	-	-	tr	-
Bergamotene <*beta*-, *trans*->	1483	1485	-	-	-	tr	-
Selinene <*beta*->	1492	1488	-	0.1	-	-	tr
allo-Aromadendr-9-ene	1489[a]	1488	-	0.1	-	-	tr
Selinene <*delta*->	1489	1491	-	tr	-	-	tr
Valencene	1494	1495	-	-	-	tr	-
Viridiflorene	1491	1496	-	1.2	-	-	0.2
Bicyclogermacrene	1497	1496	-	-	tr	-	-
Muurolene <*alpha*->	1497	1500	tr	0.1	-	-	tr
Bisabolene <(*Z*)-, *alpha*->	1503	1503	-	-	-	tr	-
Bisabolene <*beta*->	1508	1510	-	-	-	0.5	tr
Cadinene <*gamma*->	1512	1515	tr	tr	-	-	tr
Cadinene <*delta*->	1518	1523	-	1.7	tr	tr	0.2

Calamenene <*trans*->	1527	1525	0.1	-	-	-	tr
Cadina-1,4-diene <*trans*->	1536	1533	-	tr	-	-	-
Calacorene <*alpha*->	1544	1545	tr	tr	-	-	tr
Bisabolene <(*E*)-, *alpha*->	1540	1545	-	-	-	tr	-
Elemol <a*lpha*->	1546	1550	-	-	tr	-	-
Longipinanol <*epi*->	1558	1553	tr	tr	-	-	tr
Calacorene <*beta*->	1564	1564	-	tr	-	-	-
Palustrol	1568	1568	-	tr	-	-	tr
4-Dehydroviridiflorol	1572[a]	1572	-	tr	-	-	-
Spathulenol	1576	1578	-	0.1	tr	tr	tr
Caryophyllene oxide	1587	1584	1.2	tr	tr	tr	tr
Globulol	1585[a]	1585	-	0.2	-	-	tr
Viridiflorol	1594	1593	-	0.1	-	-	tr
Cubeban-11-ol	1599	1595	-	0.1	-	-	tr
Rosifoliol	1609	1604	-	tr	-	-	-
Humulene epoxide II	1613	1609	0.1	tr	-	-	-
5-Guaiene-11-ol	1619	1624	-	tr	-	-	-
Epicubenol	1631	1629	tr	0.1	-	-	tr
Eudesmol <*gamma*->	1632	1633	-	-	tr	-	-
Muurola-4,10(14)-dien-1-beta-ol	1632	1639	-	tr	-	-	-
Cadina-1(10),4-dien-8-alfa-ol	1637[a]	1640	-	-	tr	-	-
Cubenol	1649[a]	1644	-	tr	-	-	-
Muurolol <*alpha*->	1651	1647	-	tr	-	-	-
Himachalol	1650	1652	-	-	tr	-	-
Eudesmol <*alpha*->	1652	1655	-	-	tr	-	-
Cadin-4-en-10-ol	1659	1658	-	-	-	tr	-
Calamenen-10-ol <*cis*->	1663	1661	tr	tr	-	-	-
Calamenen-10-ol <*trans*->	1670	1670	tr	tr	-	-	-
Cadalene	1677	1677	-	tr	-	-	-

[a] (40)

tr: trace (<0.1%), -: not detected

LRI_{lib}: linear retention index reported in the reference library

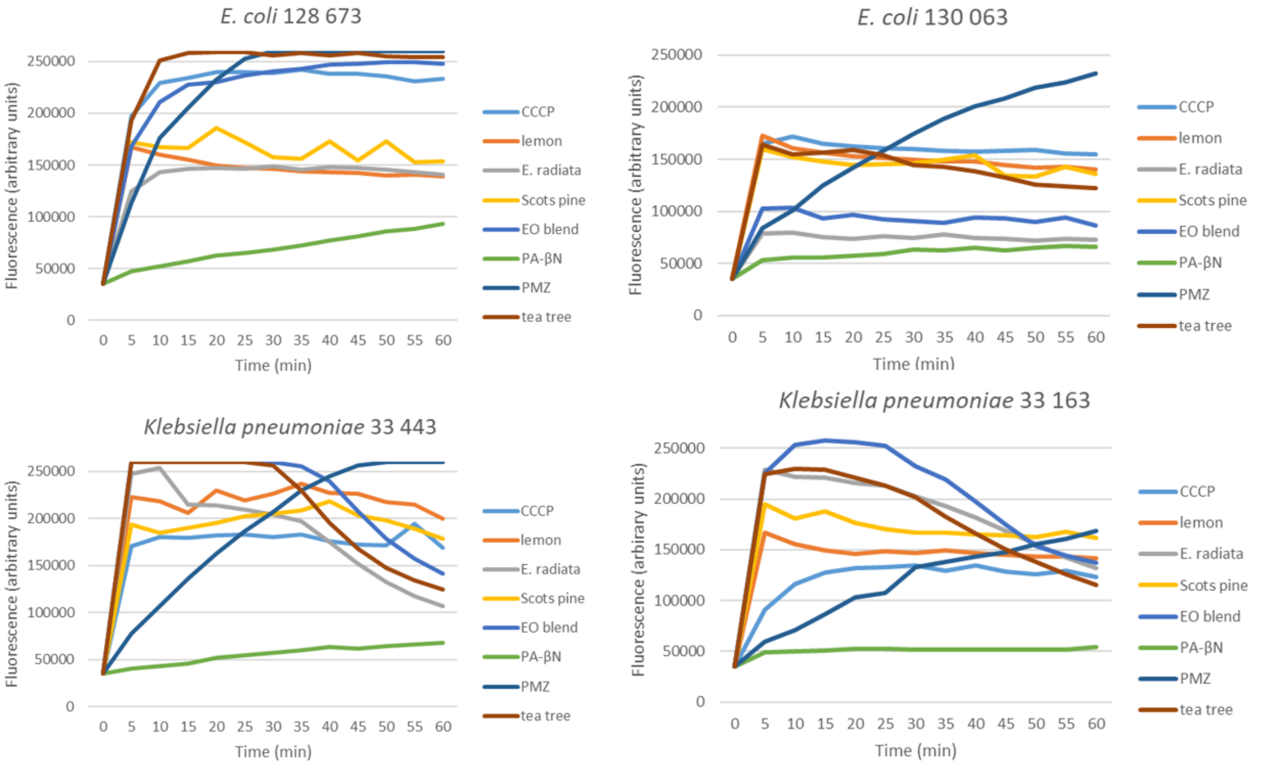

Figure S1. Time-course ethidium bromide (EB) fluorescence profiles of the tested essential oils and positive controls in *E. coli* 128673, *E. coli* 130063, *K. pneumoniae* 33443, and *K. pneumoniae* 33163 during the 60-min accumulation assay. Fluorescence is expressed in arbitrary units.
